# Ultrasound or MRI in the Evaluation of Anterior Talofibular Ligament (ATFL) Injuries: Systematic Review and Meta-Analysis

**DOI:** 10.3390/diagnostics13142324

**Published:** 2023-07-10

**Authors:** Gabriele Colò, Bianca Bignotti, Giacomo Costa, Alessio Signori, Alberto Stefano Tagliafico

**Affiliations:** 1 Orthopedic Section, National Hospital of SS. Antonio and Biagio and C. Arrigo, 15121 Alessandria, Italy; gabriele.colo@yahoo.it; 2 Department of Radiology, IRCCS-Ospedale Policlinico San Martino, 16132 Genova, Italy; alberto.tagliafico@unige.it; 3 Radiology Section, Department of Health Sciences (DISSAL), University of Genova, 16126 Genova, Italy; giacomorcg@gmail.com; 4 Biostatistics Section, Department of Health Sciences (DISSAL), University of Genova, 16126 Genova, Italy; alessio.signori@medicina.unige.it

**Keywords:** anterior talofibular ligament, ankle ultrasound, meta-analysis

## Abstract

Objectives: Ankle sprains represent the second most common cause of emergency department access for musculoskeletal injury and lateral ankle ligament complex tears account for 850,000 cases annually in the United States with a relapse rate of 70%. Clinical examination is limited due to its subjectivity and the difficulty of identifying a specific involvement of the ligament; therefore, US and MRI are frequently requested. Therefore, the goal of this study is to analyze the available literature on the use of ultrasound (US) and magnetic resonance imaging (MRI) to diagnose injuries to the anterior talofibular ligament (ATFL) with a meta-analytic approach. Methods: According to PRISMA (Preferred Reporting Items for Systematic Reviews and Meta-analyses) guidelines, all studies regarding the diagnostic accuracy of ultrasound and magnetic resonance imaging ATFL injuries were searched and assessed. The data were obtained from two independent reviewers with 12 and 3 years of experience in meta-analysis. A QUADAS-2 (Quality Assessment of Studies of Diagnostic Accuracy Studies) checklist was carried out to assess the risk of biases. From the selected studies, the sensitivity, specificity, and accuracy data were extracted. Results: Nine studies were included. The results of the meta-analysis demonstrate a greater sensitivity for ultrasound [96.88 (95% CI: 94–99) (fixed effects); 97 (95% CI: 94–99) (random effects)] compared to MRI [88.50 (95% CI: 85–91) (fixed effects); 86.98 (95% CI: 77–94) (random effects)], *p* < 0.05. The result of this meta-analysis shows that the less expensive diagnostic technique is also the most sensitive for the diagnosis of ATFL tears. Ultrasound articles resulted to have non-heterogeneity [(*p* = 0.2816; I° = 21.4607%)]. Conclusion: This meta-analysis demonstrates that US appears to be a highly sensitive diagnostic technique for diagnosing tears of the ATFL. Compared to MRI, the sensitivity of US result was higher.

## 1. Introduction

Ankle sprains affect 1 in 10,000 people every day. They represent the second most common cause of emergency department access after acute low back pain [1,2]. The most common mechanism of ankle sprain is an inversion with the foot in plantar flexion [3]. Lateral ankle ligament complex tears are the most common injury in sports, with 850,000 cases/year in the United States and a relapse rate of 70% [4,5]. The lateral ligament complex consists of three distinct ligaments: the anterior talofibular ligament (ATFL), the calcaneofibular ligament (CFL) and the posterior talofibular ligaments (PTFL) [6]. The ATFL is the weakest and about two-thirds of ankle sprains are related to isolated tears of the ATFL. Tears occur more often at the enthesis of the fibula than at the level of the talar insertion [7]. In addition, damage to the ATFL is the most common cause of chronic ankle instability [8]. Several techniques can be used to diagnose tears to the ATFL to complete physical examination: X-rays, magnetic resonance imaging (MRI), arthrography, arthrometry and ultrasound (US) [9]; however, US and MRI are the most used in clinical practice for first level assessment. Although the physical examination is essential for the clinician, with the anterior drawer test, the talar tilt test and the inversion or eversion stress test [10], it is limited due to its subjectivity and the difficulty of identifying a specific involvement of the ligament and assessment of the degree of instability [7,9,10,11,12,13]. The arthroscopic investigation may be very useful for diagnosis, also allowing an accurate classification of the lesion, but this requires hospitalization and anesthesia and exposes the patient to peri- and post-operative risks [14].

Both US and MRI have reported more than 90% accuracy in diagnosing ankle injuries, including tears to the ATFL [15]. MRI is an expensive and complex exam [16,17,18] compared to US. US, even if it is an operator-dependent technique, is cheaper, widely available and fast. US offers the possibility of having dynamic evaluation (stress ultrasound) and comparison with the healthy contralateral side [19,20,21,22,23]. Both US and MRI could be used to assess ATFL, but a direct comparison between the two techniques with a meta-analysis is missing in the literature. Therefore, the objective of this study is to compare US and MRI to diagnose tears to the ATFL with a meta-analysis to verify which could be the most appropriate to assess the ATFL.

## 2. Material and Methods

The PRISMA (Preferred Reporting Items for Systematic Reviews and Meta-analyses) guidelines were followed [24]. According to the PICOS approach, the questions relevant to the purpose are patients—symptomatic over 18 years of age; technique (intervention)—ultrasound and magnetic resonance; compare (comparison)—images obtained; result (outcome)—raw data: true negatives, false negatives, true positives and false positives, with results based on arthroscopy as a reference diagnostic study; study type—test of diagnostic accuracy.

### 2.1. Search Strategy

All the most relevant studies were identified regarding the diagnostic accuracy of ultrasound and magnetic resonance imaging ATFL tears. The data were obtained from two independent reviewers with 12 and 3 years of experience in meta-analysis (A.T. and G.C.) using major medical databases: PUBMED (http://www.pubmed.org (accessed on December 2021)), Embase (http: //www.embase.com.proxy.medlib.iupui.edu/search (accessed on December 2021), ISI Web of Science (http://apps.webofknowledge.com (accessed on December 2021)), SpringerLink, ScienceDirect and Cochrane library (http://www.thecochranelibrary.com (accessed on December 2021)), until 1 January 2022. A manual review of the reference sources to integrate the initial research with other studies was done. All citations of the selected articles were checked to find additional relevant elements. It was not considered necessary to contact the authors to ask for additional data. The keywords used in the research by the authors were: ultrasonography, magnetic resonance imaging, anterior talofibular ligament and ankle.

### 2.2. Inclusion Criteria

Studies meeting the following criteria were included:

Patients over 18 years of age with symptoms, or suspected diagnosis, of ATFL.

Diagnostic tools used: US or MRI.

Studies that used US or MRI to diagnose ATFL with appropriate reference standards (arthroscopy or other diagnostic techniques).

No limitations regarding the year of publication of the article.

Availability or ability to adequately extract at least a couple of the absolute numbers of true-negative and false-positive or true-positive and false-negative results. To include true-negative, false-negative, true-positive, and false-positive results in a meta-analysis, all four data must be available.

We included studies published in English only.

### 2.3. Exclusion Criteria

We excluded articles that did not provide data on the diagnosis of anterior talar ligament injury using US or MRI; ex vivo studies or biomechanical experiments; meta-analysis or review; and case reports.

We also excluded studies that provided data on tears of the lateral ligament complex of the ankle using US and MRI but not specifically in relation to the ATFL.

The two authors (A.T. and G.C.) independently examined the titles and abstracts of the identified articles to evaluate their content and their relevance to the research objective; then, the complete text was evaluated. Disagreements that arose during any stage of the selection process were resolved by consensus. If no agreement was found, an expert clinician (B.B.) was asked to resolve the disagreements. If the selection of an author is uncertain about any issue, this is resolved by discussing it or rereading the text. The authors A.T. and B.B. have more than 12 and 7 years of experience in musculoskeletal imaging and evidence-based medicine, both are active members of international scientific societies dealing with musculoskeletal disorders and one (A.T.) has been appointed with ESSR (European Society of Musculoskeletal Radiology) Diploma. Another author (G.C.) has more than 10 years of experience in surgery and clinical practice in the ankle and foot. The three authors have extensive experience in meta-analysis.

### 2.4. Data Extraction

The two authors (A.T. and G.C.) and a graduate student (G.C.) independently extracted data from suitable studies. Discrepancies were resolved by consensus. When available, the following data were extracted from the selected studies: first author, year of publication, type of imaging technique, number of patients (divided into groups in the case of controlled studies), mean age of patients (years), design of the study (prospective or retro-prospective), reference standard, classification and type of lesion, mean study duration (years), number of true-positive (TP), false-positive (FP), false-negative (FN) and true negative (TN). Categorical data are expressed in the number of cases or percentages. Continuous variables were reported as mean and standard deviation (SD) or mean and range. The level of evidence (LOE) of the studies was assigned based on OCEBM (Oxford Centre Evidence-based Medicine) (* OCEBM Levels of Evidence Working Group, “The Oxford Levels of Evidence 2”: http://www.cebm.net/index.aspx?o=5653 (accessed on 5 March 2022)). Studies that did not report specific variables were excluded.

### 2.5. Risk of Bias

The quality assessment of the selected studies was independently assessed by the two authors (A.T. and G.C.) and the graduate student (G.C.) using the QUADAS-2 (Quality Assessment of Studies of Diagnostic Accuracy Studies) checklist, which evaluates four fields: patient selection, test evaluated in the study, reference standards, and diagnostic times. Each domain is rated as the risk of bias and the first three as applicability. The three authors then discussed the result of their quality assessments. Disagreements were resolved with consensus. The results of the quality assessment were recorded in a QUADS-2 form, downloadable from the web page: http://www.bris.ac.uk/quadas/quadas-2 (accessed on 10 May 2022).

### 2.6. Data Synthesis and Statistical Analysis

From the selected studies, the sensitivity, specificity, and accuracy data were extracted. A funnel plot for each diagnostic technique (US or MRI) was done to assess publication biases. For smaller studies with fewer patients, a Galbraith plot was made. The analysis was carried out separately in two subgroups, depending on the diagnostic technique (US or MRI). A forest plot was produced showing the sensitivity and specificity values with the corresponding confidence intervals (CIs). *p* values of 0.05 were considered statistically significant. The Galbraith plot was used to assess heterogeneity and detect potential outliers, and the forest plot was used to report a series of central values and their confidence intervals in a graphic manner so that they can easily be compared. In the forest plot, the central values are represented by markers and the confidence intervals by horizontal lines. The I^2^ statistic was used to describe the percentage of variation across studies that is due to heterogeneity rather than chance. I^2^ = 100% × (Q − df)/Q. I^2^ is an intuitive and simple expression of the inconsistency of studies’ results which does not inherently depend upon the number of studies considered. Statistical analysis was carried out under the supervision of a professional statistician (A.S.). We used statistical software (STATA MP, StataCorp 4905 Lakeway Drive, College Station, Texas 77845 USA 800-STATA-PC and MedCalc Version 19.1.2 64 bit).

## 3. Results

The search process identified *n* = 568 articles, *n* = 527 after excluding duplicates. *n* = 449 were excluded after reading the title and *n* = 29 after reading the abstract. Of these, *n* = 49 and *n* = 40, we excluded after a careful reading of the complete text: *n* = 12 not in the English language; *n* = 3 performed in non-human; *n* = 3 case reports; *n* = 2 in vitro studies; *n* = 5 reviews; *n* = 1 done pediatric patients; and *n* = 13 without data necessary to extract the sensibility.

**Figure 1:**
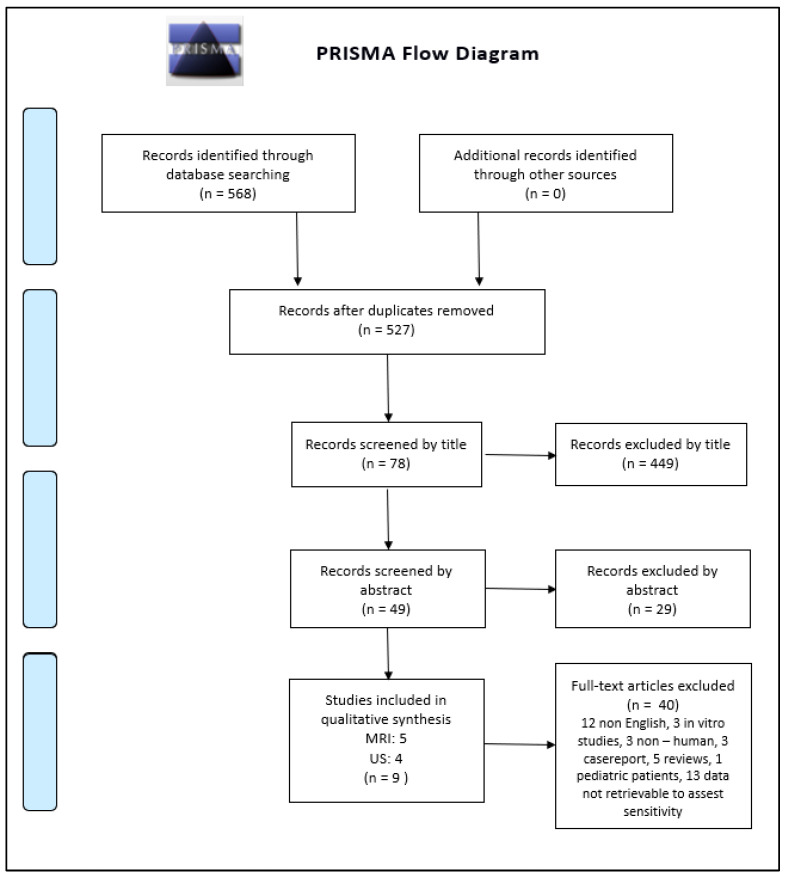
Prisma flowchart.

**Figure 2:**
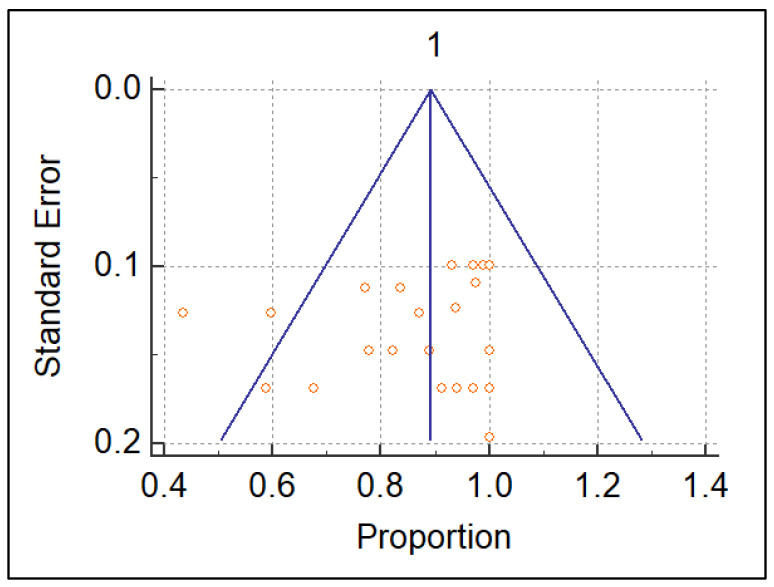
Funnel plot of every study considered in the meta-analysis.

**Figure 3:**
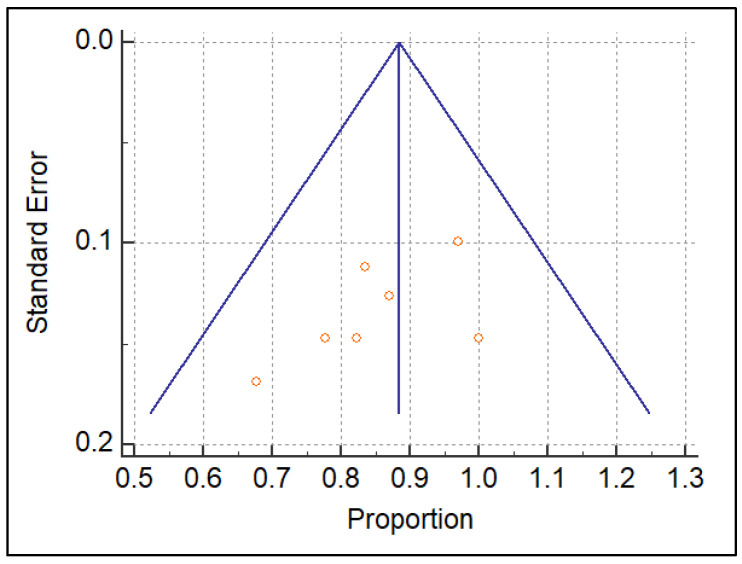
Funnel plot of MRI studies.

**Figure 4:**
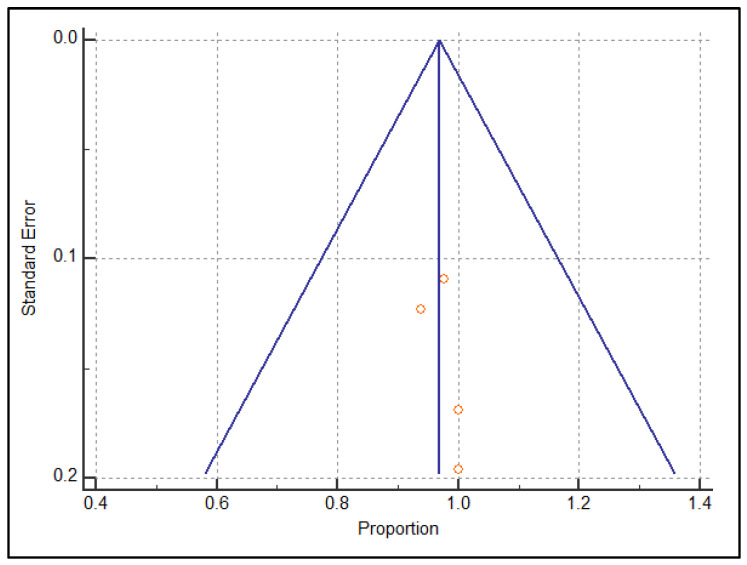
Funnel plot of US studies.

**Figure 5:**
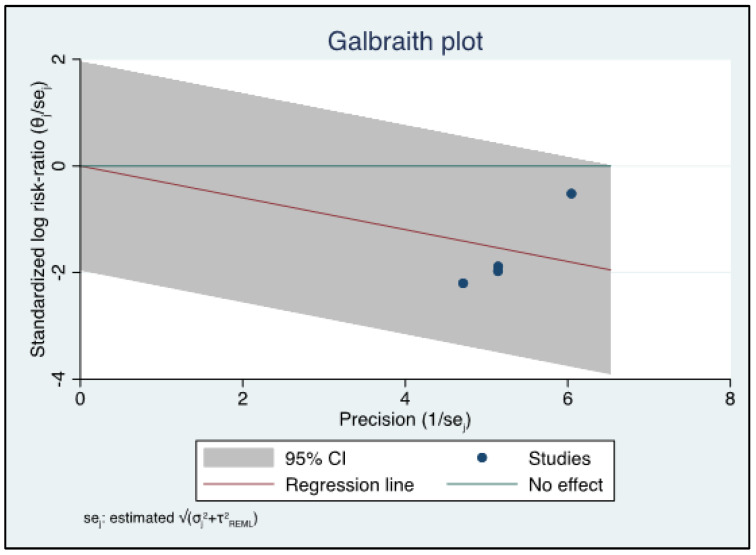
Galbraith plot for smaller studies.

At the end of our review process, we considered *n* = 4 studies regarding US and *n* = 5 dealing with MRI, for a total of *n* = 9 articles suitable for the meta-analysis. The detailed flowchart is reported according to PRISMA in Figure 1. The systematic review finally included *n* = 6 retrospective and only *n* = 3 prospective studies: *n* = 2 for US [15,25] and one for MRI [26]. The studies are relatively recent, performed between 2010 and 2021, with many recent studies including MRI. The studies are all from non-European countries, with *n* = 7 Asian out of *n* = 9 total (3 Korea, 2 China [15], 1 Japan, 1 Malaysia). The population on which the individual studies were performed ranged from *n* = 25 patients [27] to *n* = 101 patients [28]. The meta-analysis was finally performed on *n* = 9 studies reporting US or MRI sensitivity [15,16,17,18,19,20,21,22,23,24,25,26,27,28,29,30,31]. The characteristics of the nine included studies are shown in Table 1. The QUADAS-2 shown in Table 2 was used as a tool to assess the methodological quality of the included articles. The reference standard for *n* = 7 out of *n* = 9 studies was arthroscopy, while for Gun et al. [25] we refer to MRI and for Singh et al. [32] we refer to US. A heterogeneity assessment was performed on all papers (*p* < 0.0001 I° = 91.5379%) for every study; see the funnel plot in Figure 2. Only two of the studies considered were beyond the limits. The funnel plot and the heterogeneity study were also obtained separately for US (Figure 3) and MRI (Figure 4). The studies regarding US did not show any difference in results, with a null heterogeneity test (*p* = 0.2816; I° = 21.4607%). MRI studies, on the other hand, showed heterogeneity between the results (*p* < 0.0001; I° = 85.72%). For the smaller population studies (<50 patients), a Galbraith plot was made showing the studies that were within the confidence intervals (Figure 5).

**Figure 6:**
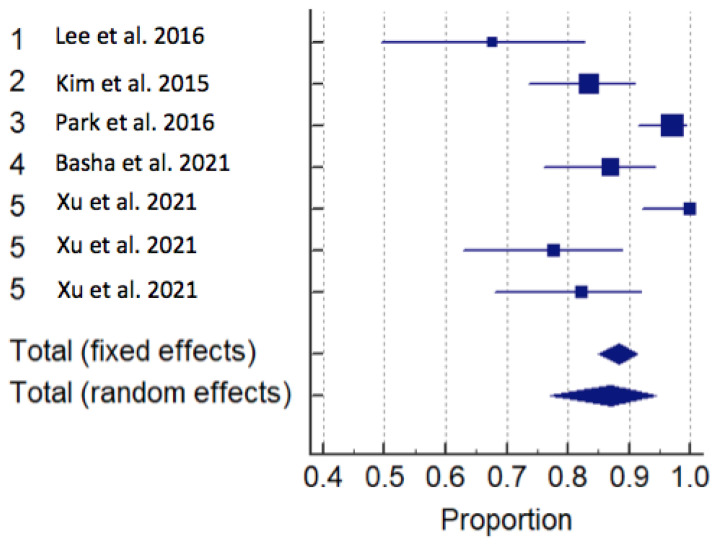
Forrest plot of magnetic resonance imaging studies for sensitivity [11,26,28,29,30].

**Figure 7:**
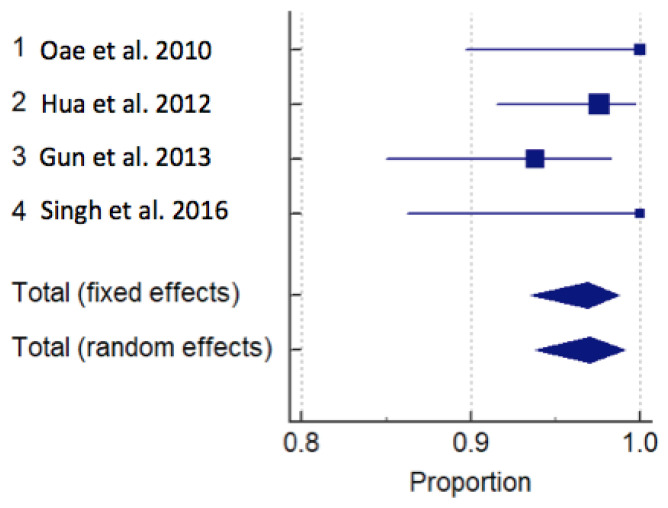
Forrest plot of ultrasound studies for sensitivity [15,25,27,31].

Sensitivity was obtained from all nine 9 articles, 4 US and 5 MRI. The meta-analysis showed that the sensitivity for the diagnosis of ATFL lesions using US is 96.88 (95% CI: 94–99) (fixed effects) and 97 (95% CI: 94–99) (random effects) and for MRI it is 88.50 (95% CI: 85–91) (fixed effects) and 86.98 (95% CI: 77–94) (random effects) *p* < 0.05. Table 3 reports data on sensitivity and specificity. Accuracy for US and MRI were, respectively: 93.50 (95% CI: 61.73–99.10) and 92.66 (95% CI: 87.08–98.24). A forest plot for MRI and US is represented in Figure 6 and Figure 7.

Study number 5 has different values for sprains, partial tears and complete tears.

## 4. Discussion

The main result of this meta-analysis is a demonstration of a greater sensitivity for US at 96.8% compared to MRI at 88.5% for the evaluation of ATFL lesions.

To diagnose an ATFL injury or tear, in addition to the clinical examination, an imaging exam is required. Indeed, the clinical examination is often unable to accurately diagnose ATFL tears. Stress radiography correlates little with real ligament damage; arthroscopy, a diagnostic and therapeutic standard, is invasive and, therefore, not of first choice. For this reason, the most appropriate and used methods are US and MRI. Among these, there is an important difference in availability, time of examination and costs, which weigh on patient and healthcare management costs [19,20,21,23]. “One limitation of US is that it remains an operator-dependent technique but it offers some advantages over other imaging techniques, in particular the possibility of having dynamic images (stress US) and the possibility to quickly compare the healthy contralateral side” [20]. Stress US means that applying a gentle force to determine valgus stress of the talofibular joint it is easier to assess the ATFL when torn. MRI is much more expensive but can give stable, reproducible, non-operator-dependent data that can be interpreted by more experts even after imaging acquisition, the so-called “second-look” evaluation. In addition, a strength of the articles dealing with US is their non-heterogeneity [(*p* = 0.2816; I° = 21.4607%)], whereas a weakness of US studies is the increased risk of bias compared to studies without an arthroscopic reference standard. Finally, studies in US had fewer patients and patients were older than those included in MRI studies. In addition, there is a lack of studies with direct comparison between US and MRI and in several studies, the reference standard is not always the same introducing potential biases.

In our long-lasting experience in musculoskeletal imaging and clinical practice, almost every patient with a possible ATFL tear will undergo imaging with US or MRI. In our clinical practice, we follow an evidence-based approach as suggested by ESSR guidelines that we contributed to develop [21,22]. Indeed, ATFL evaluation with US has an evidence level of A, which is the maximum value, with a complete consensus among experts. Given these premises, MRI should be considered not necessary for ATFL evaluation because MRI would not add any information to the US examination. However, we use MRI when an ATFL lesion could clinically be associated with other osseous or cartilaginous lesions not amenable to US examination. In addition, from the anatomical point of view, the ATFL has a very superficial location taking advantage of the higher spatial resolution of new US high-frequency probes (up to 18-MHz for routine clinical practice) compared to MRI.

The strengths of the articles on MRI are the presence of studies with minimal risk of bias, the largest number of patients and recent articles with the use of modern machinery such as 3 Tesla MRI and 3D fast spin echo sequences. Even with these notes in favor, the results showed heterogeneity between studies (*p* < 0.0001; I° = 85.72%) dealing with MRI.

Some more recent and larger works concerning US as a diagnostic technique for tears of the ATFL do not report useful data to extract its sensitivity but demonstrate its appropriateness through valid classification systems, which can be correlated with appropriate therapeutic interventions (95% of cases) [33]. An article that examines a pediatric population, a criterion that excludes it from our meta-analysis, also reports excellent US sensitivity [34].

This study has several limitations. At first, the reference standard was not always arthroscopy; indeed, arthroscopy was the reference standard for *n* = 7 out of *n* = 9 studies, whereas for Gun et al. [25] it was MRI and for Singh et al. [32] it was US. We underline that MRI as a reference for assessing the accuracy of US vs. MRI is questionable, and vice versa. The issue of a lacking arthroscopy as the gold standard in a few studies is relevant; however, it is quite difficult to obtain surgery in all patients with ATFL lesions. It is possible that patients without arthroscopy following radiological imaging had less severe lesions than patients who underwent arthroscopy following imaging. Another limitation of ultrasound as a diagnostic tool for ATFL tears is its well-known operator dependency. The quality and accuracy of ultrasound images can vary according to the operator’s experience and skills in positioning the probe and interpreting the acquired images. We do know if standard protocols were used to perform the US, as suggested by several scientific societies. Therefore, proper training and expertise are crucial to ensure reliable and accurate results when using ultrasound. In addition, US diagnostic accuracy for ATFL tears can be limited by pain. Dynamic evaluation of ATFL requires the patient to tolerate and follow the operator’s instructions during the application of gentle force to induce valgus stress on the talofibular joint. These movements are limited in case of acute pain or limited mobility, compromising the accuracy of the ultrasound examination. From a clinical perspective, we remember that US has limited capabilities in visualizing deep structures such as the intraarticular part of the ATFL. US may not offer comprehensive information when deep or associated injuries, such as osteochondral lesions or occult fractures, are suspected clinically. In such patients, magnetic resonance imaging (MRI) might be preferred for better visualization of deep and bony structures. Regarding costs and time considerations, magnetic resonance imaging (MRI) is more expensive than ultrasound. The acquisition and maintenance of MRI equipment and dedicated personnel can significantly increase costs for healthcare facilities, potentially impacting the accessibility and availability of the examination for patients. Another benefit of US could be that it is more cost-effective than MRI in most countries, even in low-income end emerging countries [35].

## 5. Conclusions

This meta-analysis demonstrates that US appears to be a highly sensitive diagnostic technique for diagnosing tears of the ATFL. Compared to MRI, the sensitivity of the US result was higher.

## Figures and Tables

**Table 1:** Characteristics of the 9 studies included in the final analysis.

Author	Year	Country	Design	Modality	Study Population (No. of Patients)	Mean Age (y) and Range	Classification	Diagnosis/Type of Injury	Duration of Study	Reference Standard
Lee et al. [11]	2016	Korea	R	MRI (3T)	34	29 (13–53)	Normal (1) Partial tear (22) Complete tear (11)	/	11 months	Arthroscopy
Kim et al. [29]	2015	Korea	R	MRI (1.5T)	79	34.6 (21–67)	/	Various ankle disorder	10 months	Arthroscopy
Park et al. [28]	2016	Korea	R	MRI (3T/3D FSE)	101	38.3 (10–80)	/	Chronic ankle pain Swelling Acute pain after a sprain	5 months	Arthroscopy
Basha et al. [30]	2021	Egypt	R	MRI (1.5T)	62	36.9 (17–52)	/	Acute sprain (28) Chronic ankle instability (18) Recurrent ankle sprain (15)	8 months	Arthroscopy
Xu et al. [26]	2021	China	P	MRI (3T)	45	32.1 (18–58)	/	Chronic ankle instability	14 months	Arthroscopy
Oae et al. [15]	2010	Japan	R	US (9 MHz)	34	29 (13–55)	Top (fibula) Middle Below (talus)	19 acute causes 15 chronic cause	2 years and 8 months	Arthroscopy
Hua et al. [31]	2012	China	R	US (5–17 MHz)	83	32.2 (17–57)	Tear: partial or total	Chronic ankle instability, impingement syndrome, osteochondral lesions, arthritis, and others	12 months	Arthroscopy
Gün et al. [25]	2013	Turkey	P	US (7.5 MHz)	65	34 (18–72)	Normal Abnormal	Inversion-type ankle injury	12 months	MRI
Singh et al. [27]	2016	Malaysia	R	US (5–13 MHz)	25	34 (18–60)	Healthy Tear injury Thickened injury	High ankle sprain	/	US

P = prospective, R = retrospective, / sign means: not available.

**Table 2:** QUADAS-2: overall risk of bias for each of the domains of patient selection, index test, reference standard, flow and timing.

		Patient Selection	Index Test (MRI or US)	Reference Standard	Flow and Timing
Lee et al. [11]	2016	+	+	+	+
Kim et al. [29]	2015	+	+	+	+
Park et al. [28]	2016	+	+	+	+
Basha et al. [30]	2021	+	+	+	+
Xu et al. [26]	2021	+	+	+	+
Oae et al. [15]	2010	+	+	+	+
Hua et al. [31]	2012	+	+	+	+
Gün et al. [25]	2013	+	+	?	?
Singh et al. [27]	2016	+	+	-	-

Yes (+), No (-), Unclear (?).

**Table 3:** Results for the sensitivity and specificity.

Study	Sample Size	Proportion (%) (Sensitivity)	95% CI
US SENSITIVITY			
Total (fixed effects)	207	96,884	93,551 to 98,783
Total (random effects)	207	97,018	93,802 to 99,090
MRI SENSITIVITY			
Total (fixed effects)	411	88,501	85,047 to 91,397
Total (random effects)	411	86,978	77,162 to 94,344
US SPECIFICITY			
Total (fixed effects)	207	88,396	83,287 to 92,386
Total (random effects)	207	84,447	55,235 to 99,396
MRI SPECIFICITY			
Total (fixed effects)	411	81,375	76,717 to 85,452
Total (random effects)	411	82,547	66,808 to 93,975

## Data Availability

Data sharing is not applicable to this article.
